# Hygiene

**Published:** 1890-09

**Authors:** 


*Hygiene.* By LOUIS C. PARKES, M.D. Second Edition. London: H. K. LEWIS. 1890.

It must have been highly satisfactory to Dr. Parkes to have had so soon to prepare a second edition of his book, which we noticed favourably upon its first appearance last year. The usefulness of the work has been increased by correction and addition.

